# A retrospective case-series of influence of chronic hepatitis B on synchronous liver metastasis of colorectal cancer

**DOI:** 10.3389/fonc.2023.1109464

**Published:** 2023-02-23

**Authors:** Lin Zhu, Piqing Gong, Ye Liu, Yunjie Shi, Wenqiang Wang, Wei Zhang, Zhiqian Hu, Xinxing Li

**Affiliations:** ^1^ Department of General Surgery, Tongji Hospital, Medical College of Tongji University, Shanghai, China; ^2^ Department of Anorectal Surgery, Changzheng Hospital, Naval Medical University (Second Military Medical University), Shanghai, China; ^3^ Department of Blood Transfusion, Changzheng Hospital, Naval Medical University (Second Military Medical University), Shanghai, China

**Keywords:** chronic hepatitis B, synchronous liver metastasis, colorectal cancer, HBV, liver cirrhosis

## Abstract

**Main point:**

Our retrospective analysis of a large number of cases found in patients with primary colorectal cancer (CRC) carrying positive HBsAg inhibited the occurrence of synchronous liver metastases (SLM). However, liver cirrhosis caused by non-HBV factors promoted the occurrence of SLM.

**Objectives:**

This study aimed to investigate the effect of HBV on the occurrence of synchronous liver metastases (SLM) of colorectal cancer (CRC).

**Methods:**

Univariate and multivariate analyses were used to analyze the influence of clinical parameters on the occurrence of SLM.

**Results:**

A total of 6, 020 patients with primary CRC were included in our study, of which 449 patients carrying HBsAg(+) accounted for 7.46%. 44 cases of SLM occurred in the HBsAg(+) group, accounting for 9.80%, which was much lower than 13.6% (758/5571) in the HBsAg(-) group (X=5.214, P=0.022). Among CRC patients with HBsAg(-), the incidence of SLM was 24.9% and 14.9% in the group with high APRI and FIB-4 levels, respectively, which were significantly higher than that in the compared groups (12.3% and 12.5%, all P<0.05). Compared with the control group, female patients, late-onset patients, and HBV-infective patients had lower risks of SLM (HR=0.737, 95%CI: 0.614-0.883, P<0.001; HR=0.752, 95%CI: 0.603-0.943, P=0.013; HR=0.682, 95%CI: 0.473-0.961, P=0.034).

**Conclusions:**

The carriage of HBsAg(+) status inhibited the occurrence of SLM from CRC. HBV-causing liver cirrhosis did not further influence the occurrence of SLM, whereas non-HBV-factor cirrhosis promoted the occurrence of SLM. Nevertheless, this still required prospective data validation.

## Introduction

Colorectal cancer (CRC) was one of the most common malignancies of the gastrointestinal tract. CRC ranked the third in incidence and was the second leading cause of cancer-related deaths worldwide (1). CRC was also one of the most prevalent cancers in China, where the mortality rate of CRC was about 13.13/100, 000, accounting for 7.8% of the total number of deaths among patients with malignant tumors (2). Recurrence and distant metastasis were the two main factors affecting the survival of CRC (3). The most common target organ of distant hematogenous metastases of CRC was liver (4). Colonic venous blood converged into the hepatic portal vein through the superior and inferior mesenteric veins, respectively. It was the anatomical structure and portal circulation pathway that made the liver the preferred site for distant metastases. About 15-25% of patients suffered from synchronous liver metastasis (SLM), while another 15-25% developed metachronous liver metastases postoperatively (5). Ultimately, approximately 50% of patients developed liver metastases at some point throughout the course of their disease (6). Patients with untreated liver metastases had a median survival of only 6.9 months (7). Although complete surgical resection was provided, the median survival period was less than 35 months (7). Obviously, CRC liver metastasis was a thorny problem in clinical diagnosis and treatment.

According to WHO, 1.1 million people were newly infected with chronic hepatitis B virus (HBV) in 2017 (7). As of 2016, there were 267 million chronic hepatitis B (CHB) infections worldwide and 1.4 million deaths from viral hepatitis, 96% of which were caused by hepatitis B and C viruses (7, 8). China conducted the first national hepatitis seroepidemiological survey in 1992, according to which approximately 120 million people in China carried HBsAg(+), and nearly 300, 000 died from HBV infection each year (9). CRC patients with comorbid chronic HBV infection were also more common in clinical practice. However, whether CHB promoted or suppressed synchronous liver metastasis was controversial. Some concluded that the incidence of liver metastases was reduced in CRC patients with concomitant CHB infection (10). Obviously, CHB infection had a suppressive effect on liver metastases, but the sample size included was small. Although others thought that CRC with concomitant CHB infection promoted liver metastases (11, 12), the inclusion criteria were controversial.

Thus, in our study, a retrospective analysis of a large sample was conducted to explore the effect of HBV on SLM of CRC, aiming to clarify a clear connection between HBV and SLM in CRC, to provide a basis for further clinical and basic research on CRC liver metastasis.

## Patients and methods

### Clinical information

A total of 6, 020 consecutive patients with CRC who were admitted to Shanghai Changzheng Hospital from July 2010 to June 2021 were selected. The study was approved by the ethics committee of Shanghai Changzheng hospital. The clinical data collected included age, gender, tumor metastasis, HBV carrier status, blood type, CEA, CA199, AFP, primary tumor location, primary tumor diameter, tissue type, degree of differentiation, and depth of tumor invasion. Locations of the primary tumor were divided into left colon, right colon, and rectum. Diameter of the primary tumor was divided into ≤3 cm and >3 cm according to the size. Tissue types were divided into adenocarcinoma and other types (carcinoid, signet ring cell carcinoma, mucinous adenocarcinoma, etc.). Degrees of differentiation were divided into undifferentiated-poor differentiation and medium-well differentiation. Depths of invasion were divided into T_1-2_ group and T_3-4_ according to the TNM staging standard formulated by AJCC. Each patient selectively underwent X-ray, abdominal B-ultrasound, chest CT, abdomen CT, abdominal MRI, or PET-CT according to the diagnosis and treatment needs.

### Inclusion and exclusion criteria

#### Inclusion criteria

(1) Colonoscopy biopsy or surgical pathology was performed (2). Specific HBV carrier statuses were recorded, such as HBsAg, HBsAg, HBsAg, HBeAb, and HBcAb (3). The diagnosis of distant metastases was issued with clear imaging data support, such as B-ultrasound, CT, MRI, or PET-CT.

#### Exclusion criteria

(1) Benign colorectal diseases: colorectal polyps, familial polyposis, ulcerative colitis, Crohn’s disease. (2) Other diseases of the colorectum: neuroendocrine tumors, lymphoma, intestinal tuberculosis, typhoid fever, intestinal amebiasis, Intestinal schistosomiasis, etc. (3) Serious lack of clinical data: such as age, gender, primary tumor location, SLM information, etc. (4) Combined with other archenteric malignant tumors. (5) Patients who had undergone surgery or radiotherapy and chemotherapy at the time of admission. (6) Patients who had lung metastasis and concomitant metastases of other organs, such as liver metastasis.

#### Diagnostic criteria

1. CRC: all patients included in the study had a definite diagnosis of CRC. Patients who underwent surgery had a complete postoperative pathology report. Patients with advanced stage or metastases who did not undergo surgery were diagnosed by colonoscopy biopsy. 2. Liver cirrhosis: Aspartate aminotransferase-to-platelet ratio index (APRI) and Fibrosis 4 Score (FIB-4) were used as an indirect indicator for the diagnosis of liver cirrhosis with the cut-off values of 0.5 and 1.45, respectively (13, 14). APRI lower than 0.5 was generally considered to exclude liver cirrhosis, and FIB-4 lower than 1.45 was generally considered to exclude liver cirrhosis (14). 3. Definition of SLM of CRC: according to international consensus (15) and the “Guidelines for the diagnosis and comprehensive treatment of liver metastases of CRC in China (2020)” (16), synchronous liver metastasis referred to liver metastases found before or at the time of diagnosis of CRC. 4. Imaging diagnosis of SLM: at least 2 or more imaging physicians with associate high title issued the corresponding diagnostic reports. The confirmation of intraoperative liver metastases should be determined by at least 2 experienced surgeons.

### Statistical analysis

SPSS 20.0 statistical software was used for statistical analysis. The numerical variables were converted into categorical variables, which were uniformly tested by the chi-square test. Univariate analysis was performed on the factors that might affect SLM, and multivariate Logistic regression analysis was performed on the statistically significant indexes. P<0.05 was statistically significant.

## Results

### Clinical characteristics

As shown in **Table 1**, a total of 6, 020 patients with primary CRC were enrolled in this study, 3810 males and 2210 females, with an age range of 14-105 years and a median age of 63.0 years. Among them, there were 449 CRC patients with HBsAg(+), accounting for 7.46%. There were 802 patients with synchronous liver metastasis in all cases, among which 44 patients with HBsAg(+) complicated with synchronous liver metastasis, accounting for 9.80% in the HBsAg(+) group; while 758 patients with HBsAg (–), accounting for 13.6% in the HBsAg (–) group. Compared with the HBsAg (–) group, the proportion of SLM was lower in the HBsAg(+) group. There was a statistical difference between the two groups (P<0.05), which suggested that HBV might inhibit the occurrence of SLM in CRC.

**Table 1:** Clinical parameters and characteristics.

Clinical parameters	Enrolled cases N=6020	HBsAg(+) N=449	HBsAg (–) N=5571	χ^2^	P value
**Gender**				3.674	0.055
male	3810	303(67.5%)	3507(63.0%)		
female	2210	146(32.5%)	2064(37.0%)		
**Age (years)**				46.304	**0.000**
<50	956	122(27.2%)	834(15.0%)		
≥50	5064	327(72.8%)	4737(85.0%)		
**Blood type**				6.273	0.180
O	1918	159(35.4%)	1759(31.6%)		
A	1919	140(31.2%)	1779(31.9%)		
B	1526	99(22.0%)	1427(25.6%)		
AB	561	47(10.5%)	514 (9.2%)		
missing data	96	4 (0.9%)	92 (1.7%)		
**CEA**				0.936	0.632
normal	3365	261(58.1%)	3104(55.7%)		
high	2596	184(41.0%)	2412(43.3%)		
missing data	59	4 (0.9%)	55 (1.0%)		
**CA199**				2.655	0.265
normal	4813	353(78.6%)	4460(80.1%)		
high	1103	84(18.7%)	1019(18.3%)		
missing data	104	12 (2.7%)	92 (1.7%)		
**AFP**				10.519	**0.012**
normal	5860	427(95.1%)	5433(97.5%)		
high	13	4 (0.9%)	9 (0.2%)		
missing data	147	18 (4.0%)	129 (2.3%)		
**Tumor location**				0.773	0.679
right colon	1380	102(22.7%)	1278(22.9%)		
left colon	1520	121(26.9%)	1399(25.1%)		
rectum	3120	226(50.3%)	2894(51.9%)		
**Tumor size (cm)**				0.942	0.642
≤3	1756	139(31.0%)	1617(29.0%)		
>3	4239	308(68.6%)	3931(70.6%)		
missing data	25	2 (0.4%)	23 (0.4%)		
**Pathological type**				0.243	0.622
adenocarcinoma	5221	386(86.0%)	4835(86.8%)		
^#^others	799	63(14.0%)	736(13.2%)		
**Differentiation**				2.320	0.313
G1-G2	342	31 (6.9%)	311 (5.6%)		
G3-G4	5389	401(89.3%)	4988(89.5%)		
missing data	289	17 (3.8%)	272 (4.9%)		
**Invasion depth**				1.686	0.430
T1-T2	1715	120(26.7%)	1595(28.6%)		
T3-T4	3929	305(67.9%)	3624(65.1%)		
missing data	376	24 (5.3%)	352 (6.3%)		
**SLM**				5.214	**0.022**
yes	802	44 (9.8%)	758(13.6%)		
no	5218	405(90.2%)	4813(86.4%)		

^#^ other types: carcinoid, signet ring cell carcinoma, mucinous adenocarcinoma, etc. P<0.05 was statistically significant. P-values less than 0.5 are marked in bold.

In order to know the published data on the effect of HBV on CRC liver metastasis in the past 20 years, we searched CRC patients in PubMed, Web of Science, and Embase with the keywords HBsAg, HBV, CRC, colon cancer, rectal cancer, and liver metastasis. We searched 13 retrospective analyses, of which 10 articles were published by Chinese scholars, 2 by Italian scholars, and 1 by Japanese scholars. Among them, 4 suggested that HBV promoted CRC liver metastasis, and 9 suggested that HBV inhibited CRC liver metastasis (**Table 2**). In studies with over 3, 000 CRC patients enrolled, HBV was believed to promote the occurrence of liver metastases. However, the definitions of liver metastases above were controversial and failed to distinguish SLM from metachronous liver metastases (**Table 2**). Even for SLM, the established criteria were inconsistent.

**Table 2:** Effects of HBV on CRC liver metastases published during 1999-2022.

Years	Nation	Cases	HBsAg+ratio	Rate of CRLM: HBsAg(+) vs HBsAg(-)	Inhibit or promote	Journal
2019	China (17)	7187	5.12%	13.40% vs. 8.54%	+	Annals of Oncology
2018	China (12)	4033	6.1%	15.57% vs. 8.60%	+	Clinical infectious diseases
2022	China (18)	3914	13.19%	16.95% vs. 13.06%	+	Scientific Report
2022	China (11)	3132	13.2%	16.5% vs. 12.7%	+	Cancer Management and Research
2014	China (19)	1413	–	9.4% vs. 23.9%	–	Hepatogastroenterology
2011	China (10)	1298	2.9%	14.2% vs. 28.2%	–	World journal of gastroenterology
2020	China (20)	884	33.60%	1.68% vs. 5.28%	–	International Journal of Colorectal Disease
2005	Italy (21)	630	9.21%	17.2% vs. 33.1%	–	Minerva chirurgica
2001	China (22)	512	14.45%	13.51% vs. 27.17%	–	American journal of surgery
2013	Italy (23)	488	6.35%	3.2% vs. 9.4%	–	Annali italiani di chirurgia
1999	Japan (24)	438	8.45%	8.11% vs. 21.20%	–	American journal of surgery
2012	China (25)	354	19.77%	2.86% vs. 16.9%	–	Hepatogastroenterology
2018	China (26)	289	12.1%	18.42% vs. 81.58%	–	Journal of Cancer

+: Promote; -: Inhibit. CRLM: CRC liver metastasis.

In addition, we also found that the status of HBsAg in CRC patients was also related to age and AFP. Early-onset CRC patients (age <50 years old) accounted for 27.2% (122/449) in HBsAg(+) group, which was more than 15.0% (834/5571) in HBsAg (–) group. Among the patients with HBsAg(+), elevated AFP levels accounted for 0.9% (4/449), higher than 0.2% (9/5571) in the HBsAg(-) group. We assumed that this was probably because the infection of HBV could cause damage to hepatic cells, leading to the elevation of AFP, which seemed not to contradict the conclusion that HBsAg(+) inhibited SLM in CRC patients. However, HBsAg status was not related to gender, blood type, CEA, CA199, tumor location, tumor size, tissue type, degree of differentiation, and depth of invasion (P>0.05) (**Table 1**).

### APRI, FIB-4 promoted SLM in non-HBsAg(+) group

We further explored the effects of e-antigen, liver cirrhosis indicators, and virus carrier status on the occurrence of simultaneous liver metastases. In 449 HBsAg(+) patients, the effects of e-antigen, liver cirrhosis index, and virus carrier status on the occurrence of SLM were analyzed. Different from a previous report (12), we did not find that e-antigen, liver cirrhosis indicators (APRI and FIB-4), and virus replication status [HBsAg/HBeAg/HBcAb(+) and HBsAg/HBeAb/HBcAb(+)] had any effect on the occurrence of simultaneous liver metastases in HBsAg(+) CRC patients (P>0.05) (**Table 3**). Interestingly, in the non-HBsAg+ group, the incidence of SLM in the high APRI and FIB-4 groups was 24.9% and 14.9%, respectively, which was significantly higher than that in the low APRI and FIB-4 groups (12.3% and 12.5%, P < 0.05) (**Table 4**), suggesting that cirrhosis or liver fibrosis may promote the occurrence of SLM in non-HBV-infected CRC.

**Table 3:** Effect of e-antigen, liver cirrhosis, and virus carrier status on CRLM in HBsAg(+) group.

Group	SLM, N (%)	No SLM, N (%)	P value
**HBeAg**			1.000
+	5(10.9%)	41(89.1%)	
-	39(9.7%)	364(90.3%)	
**APRI**			0.111
APRI high level	4(5.0%)	76(95.0%)	
APRI low level	40(10.8%)	329(89.2%)	
**FIB-4**			0.963
FIB-4 high level	22(9.7%)	204(90.3%)	
FIB-4 low level	22(9.9%)	201(90.1%)	
**Virus carrier status**			0.499
HBsAg/HBeAg/HBcAb(+)	5(11.6%)	38(88.4%)	
HBsAg/HBeAb/HBcAb(+)	21(8.4%)	228(91.6%)	
Unknown	18(11.5%)	139(88.5%)	

CRLM: CRC liver metastasis. P<0.05 was statistically significant.

**Table 4:** Effect of liver cirrhosis index on CRLM in non-HBsAg+ group.

Group	SLM, N (%)	No SLM, N (%)	P value
**APRI**			<0.001
APRI high level	148(24.9%)	446(75.1%)	
APRI low level	610(12.3%)	4367(87.7%)	
**FIB-4**			0.004
FIB-4 high level	427(14.9%)	2441(85.1%)	
FIB-4 low level	331(12.2%)	2372(87.8%)	

CRLM: CRC liver metastasis. P<0.05 was statistically significant.

### Univariate and multivariate analysis on SLM in CRC

Univariate analysis showed that gender, age, CEA, CA199, tumor location, tumor size, tissue type, degree of differentiation, depth of infiltration, and HBsAg status were factors influencing the occurrence of CRC SLM (P<0.05). Further, we found that gender, age, CEA, CA199, tumor size, tissue type, degree of differentiation, depth of invasion and HBsAg status were independent factors affecting the occurrence of SLM in CRC (P<0.05) (**Supplementary Table 1**).

Excluding groups with incomplete data on clinical parameters (CEA, CA199, tumor size, tissue type, degree of differentiation, and depth of infiltration), gender, age, and HBsAg status were independent factors influencing the occurrence of SLM (P< 0.05), while tumor location was not an independent factor (P>0.05). Compared with the control group, female patients had a lower risk of developing CRC synchronous liver metastasis (HR=0.737, 95%CI: 0.614—0.883, P<0.001). Similar results have been observed in late-onset CRC patients (HR=0.752, 95%CI:0.603—0.943, P=0.013) and CRC patients with HBsAg(+) (HR=0.682, 95%CI:0.473—0.961, P=00.034) (**Supplementary Table 5**).

### Effect of HBV on SLM in the early-onset CRC group

The above results suggested that the proportion of HBsAg(+) in early-onset CRC patients was higher, suggesting that early-onset CRC might be a suppressive factor for SLM.

Therefore, in order to further explore whether the low incidence of SLM in early-onset CRC was related to HBV infection, we investigated the effect of HBsAg status on SLM. As seen in **Supplementary Table 2**, in the early-onset CRC group, HBsAg status was not associated with the occurrence of SLM (P=0.108). Apparently, the occurrence of SLM in early-onset CRC was more closely related to exposure factors, dietary habits, body immune status, gene expression, and mutation correlation.

Similarly, **Supplementary Table 3** showed that in the early-onset CRC with HBsAg+ group, e-antigen, liver cirrhosis indicators, and virus carrier status were not associated with the occurrence of SLM.

### Effect of HBV on SLM in colon cancer

Although we found that after dividing the CRC into the left half, right half, and rectum according to the tumor location, the tumor part was not an independent factor affecting the occurrence of synchronous liver metastasis. However, after dividing CRC into colon and rectum, the rate of concurrent liver metastases from colon cancer was 15.2% (442/2458), which was higher than that in rectal cancer (10.11%, 360/3562), also being an independent factor influencing the occurrence of SLM (P<0.5), consistent with the data reported in the literature (16). Therefore, we further explored the effect of HBV on synchronous liver metastasis in colon cancer. In **Supplementary Tables 4**, **5**, we found that HBsAg status, e-antigen, APRI, and FIB-4 were unrelated to the occurrence of SLM (P>0.05). We speculated that the higher incidence of SLM in the colon might be more attributed to anatomical superior and inferior mesenteric venous reflux to the portal system, while the rectal portion returned to the inferior vena cava (body circulation).

### Effect of HBV on synchronous extrahepatic (lung) metastases

The effect of HBV on extrahepatic metastasis, especially lung metastasis, remained unclear. Our study found that the rates of synchronous lung metastases in HBsAg(+) and HBsAg(-) were 1.7% and 1.53%, respectively. There was no statistical difference between them (P=0.576) (**Table 5**). This suggested that the occurrence of synchronous lung metastases was not related to the status of HBV infection, but more probably associated with systemic blood circulation and lung microenvironment.

**Table 5:** Influence of HBsAg status on lung metastasis.

Parameters	Synchronous lung metastases, N (%)	No synchronous lung metastases, N (%)	Total	χ^2^	P value
**HBsAg**				0.313	0.576
+	6(1.7%)	443(98.3%)	449		
-	94(1.53%)	5477(98.47%)	5571		
**Total**	100(1.7%)	5920(98.3%)	6020		

P<0.05 was statistically significant.

## Discussion

Recurrence and metastasis were the leading causes of death in CRC patients (27). The liver was the most common metastatic organ of CRC (28). Resection of liver metastases was the preferred method for the treatment of CRC with liver metastases (29). However, approximately 75% of patients relapsed within 2 years (30). Due to a large number of HBV infective patients and CRC patients worldwide, so what was the relationship between HBV infection and CRLM? Before discussing the relationship between HBV and CRLM, we first defined the definition of synchronous CRLM. The Expert Group on the Treatment of Liver Metastases discussed this issue and reached a consensus (31) that SLM were referred to as simultaneously discovered liver metastases detected at the time of primary CRC tumor diagnosis. Although the classification of SLM had reached an international consensus, actually the standards in researches were not uniform. Some argued that SLM were liver metastases found at the time of CRC diagnosis or within 6 months after radical resection of the primary CRC (15). Nevertheless, if liver metastases happened within 6 months after surgery, it meant that metastases had already occurred before surgery. In the early stages of metastasis, minimal residual diseases were undetectable. Because CT only could distinguish lesions larger than 0.5 cm, while B-ultrasound only larger than 1 cm (32). In addition, the reports on the incidence of synchronous and metachronous liver metastases were controversial due to the limited sample size (21, 23). Here, we selected SLM according to the international consensus (31) that liver metastases found before or at the time of diagnosis of CRC, which could allow us to judge the occurrence of SLM more accurately.

The controversy was still ongoing regarding the impact of HBV infection on the risk of CRC liver metastases. Most studies thought that HBV infection inhibited the occurrence of CRC liver metastases (26, 33); at the same time, other few studies held an opposite view (11, 17). A retrospective study by Huo et al. (12) collected 4,033 CRC patients to conclude that concomitant chronic HBV infection significantly increased the risk of CRC liver metastases with a higher hazard risk (2.317), compared with CRC patients not infected with HBV. However, the mechanism of HBV infection promoting CRC liver metastasis was unclear. Chemokines in tumor microenvironment promoted malignant tumor metastasis through multiple mechanisms (34). CRC cells recruited specific subsets of myeloid cells to facilitate cancer cell growth in the liver through the chemokine CCL2 (35). They were combined with monocyte chemoattractant protein (MCP-1) to form MCP-1/CCR2, which promoted the growth of CRC in the animal. Once HBV infection occurred, the expression level of MCP-1 was up-regulated (36). This might hint that HBV infection facilitated liver metastasis of CRC. Also, the expressions of chemokines CCL20, CXCL6, and CXCL9/10/11 increased in HBV-infected patients, which were all related to the occurrence of CRC (36–38).

We found that HBV may inhibit SLM in CRC. We enrolled 6, 020 cases, of which 802 patients developed SLM. There were 44 cases with simultaneous liver metastasis in the HBV infective group. Compared with the HBsAg (–) group, the proportion of SLM in the HBsAg(+) group was lower (9.80% vs. 13.6%). Utsunomiya et al. (24) found that liver metastases were rare in HBV or HCV-infected CRC patients. Song et al. (22) reported that HBV-infected patients had fewer CRC liver metastases and more prolonged survival than non-HBV-infective patients. Another research showed that HBV infection and liver cirrhosis could reduce the incidence of liver metastases in CRC patients, but did not affect their survival rate. Wang et al. (25) and Qiu et al. (10) also came to a similar conclusion that HBV inhibited the SLM of CRC.

The mechanism by which HBV infection inhibited CRC liver metastasis was also still unclear. Some studies held that HBV enhanced the host’s cellular and humoral immune function after HBV entered the body. HBV replication not only enhanced the killing of cytotoxic T lymphocytes (CTL) and Kupffer cells to wipe out cancer cells, but activated cytokines such as TNF-α and INF-γ to boost the antitumor effects (10, 39). HBV infection promoted the production of cytokines such as INF-γ and IL-6 by activating Kupffer cells, CTLs, and monocytes, while INF-γ inhibited the formation of neovascularization in cancer metastases. IL-6 indirectly increased liver ECM, thereby inhibiting CRC cell metastasis or making it difficult for CRC cells to transfer to the liver for growth and proliferation (40). During the progression from CHB to cirrhosis, Kupffer cell activation led to tissue damage and even liver fibrosis, and inhibited CRC liver metastasis. Other studies reported that microRNAs silenced target genes through mRNA degradation or translation inhibition to inhibit the occurrence of liver metastasis, such as miRNA-145, Let-7, etc (41, 42). Also, tumor liver metastases were intrinsic to tumor cells and influenced by the local metastatic tumor microenvironment (43). The imbalance between matrix metalloproteinases (MMPs) and their inhibitors contributed to CRC progression and invasion (44). MMP inhibitors used to treat CRC in animal models suggested that increased expression of MMPs inhibited the colonization of chronic hepatitis-infected tumor cells and hindered colon cancer liver metastasis (45). Another possible explanation we thought was that CRC secreted CEA that could specifically bind to the CEA receptor on liver Kupffer cells so that Kupffer cells produced IL-α, IL-1β, IL-6, and TNF-α, inducing liver Sinusoidal endothelial cells to express intercellular adhesion molecules. Next, metastatic cancer cells adhered to the liver sinusoidal endothelial cells, so as not to enter the liver.

Liver cirrhosis was a common clinical chronic progressive liver disease, diffuse liver damage formed by long-term or repeated action of one or more causes (46–49). In China, most of them were post-hepatitis cirrhosis, while a few were alcoholic cirrhosis and schistosomiasis (12, 50, 51). What was the relationship between post-hepatitis cirrhosis and CRC liver metastasis? Huo et al. (12) used APRI as an evaluation index for the severity of liver cirrhosis and found that in CRC patients with positive HBsAg, patients with high APRI (>0.5) had a lower probability of developing SLM than patients with low APRI (≤0.5). Liver metastases from CRC were rarely shown in patients with liver cirrhosis, a retrospective study in the United States showed (52). However, a study in Taiwan put forward the opposite view, arguing that the risk of liver metastases in CRC patients with liver cirrhosis was underestimated, presenting that the risk of liver metastases in CRC patients with liver cirrhosis was higher (53). Nevertheless, in 449 cases of HBsAg(+) CRC patients in our study, we did not find that e-antigen and liver cirrhosis indicators (APRI, FIB-4) had any effect on the occurrence of SLM. This suggested that HBV-induced liver cirrhosis did not further affect the occurrence of SLM. There might be the following reasons we thought for the above results: 1. A better indicator of HBV replication was the level of DNA replication. 2. APRI and FIB-4 could not accurately reflect the actual degree of liver cirrhosis or liver fibrosis. 3. The information on whether patients took hepatoprotective or antiviral drugs or not was missing. But interestingly, we found that in HBsAg (–) CRC patients, the incidences of SLM in the high APRI and FIB-4 groups were 24.9% and 14.9%, respectively, which were significantly higher than those in the low APRI and FIB-4 groups (12.3% and 12.5%), suggesting that non-HBV factors in liver cirrhosis promoted the occurrence of SLM from CRC. The possible underlying mechanism was the effect of mechanical factors, such as mesenteric circulation and hepatic capillaries, which promoted liver metastasis (54). Patients with liver cirrhosis had intestinal epithelial barrier dysfunction compared with healthy subjects (55–57). In addition, vascular remodeling and tortuosity led to direct shunting of portal and arterial blood supply to the hepatic outflow tract, and eventual vessel tortuosity and slow blood flow further facilitated cancer cell seeding (57, 58). The new finding opened up new ideas for us to further study the pathogenetic mechanism of synchronous liver metastasis of CRC, but it still needed prospective data verification.

This study had the following deficiencies and limitations: 1. Status of HBV carriers. It would be more convincing to clarify the role of HBV-DNA status in tumor pathogenesis. 2. HBV treatment and outcome. Antiviral therapy duration, regimen, and outcomes also affected final clinical outcomes. Besides, many retrospective studies have not been able to investigate whether the tumor occurred or HBV infection first. 3. Different definitions of liver metastases and defects in detection methods. Different definitions of liver metastases in CRC would inevitably lead to bias in the analysis of results. Also, the resolutions and models of imaging equipment in different hospitals or different periods of the same hospital were quite different, resulting in diagnostic defects and final research bias.

In conclusion, despite the controversies shown in the review of literature, the retrospective analysis of a large number of cases in our study found that in patients with primary CRC, carrying positive HBsAg might inhibit the occurrence of SLM. As for early-onset CRC patients, it seemed that HBsAg status was not associated with the occurrence of SLM. The rate of concurrent liver metastases from colon cancer was higher than that in rectal cancer. However, HBsAg status seemed unrelated to the occurrence of SLM in colon cancer. Besides, HBV-induced liver cirrhosis appeared not to further affect the occurrence of SLM while liver cirrhosis caused by non-HBV factors promoted the occurrence of SLM. Meanwhile, it seemed that HBsAg status had no effects on the incidences of lung metastasis in CRC. These findings still required prospective data validation.

## Data availability statement

The raw data supporting the conclusions of this article will be made available by the authors, without undue reservation.

## Ethics statement

The studies involving human participants were reviewed and approved by the ethics committee of Shanghai Changzheng hospital. The patients/participants provided their written informed consent to participate in this study.

## Author contributions

LZ, PG, and YL contributed equally to this work and share first authorship. All authors contributed to the article and approved the submitted version.
