# Supplementary material for: A retrospective case-series of influence of chronic hepatitis B on synchronous liver metastasis of colorectal cancer
Source: Front Oncol. 2023 Feb 23;13:1109464. doi: 10.3389/fonc.2023.1109464 (PMC9995980; doi:10.3389/fonc.2023.1109464)
Supplement: Supplementary file 1 [file Table_1.docx]

**Supplementary table 1. Univariate and multivariate analysis on the occurrence of synchronous liver metastases in CRC**

| **Clinical parameters** | **Univariate** **analysis** | | **multivariate analysis** | |
| --- | --- | --- | --- | --- |
|  | **HR（95%CI）** | **P value** | **HR（95%CI）** | **P value** |
| **Gender** |  |  |  |  |
| male | reference |  | reference |  |
| female | 0.713(0.607-0.837) | ＜0.001 | 0.737(0.614---0.883) | **＜0.001** |
| **Age (years)** |  |  |  |  |
| <50 | reference |  | reference |  |
| ≥50 | 0.801(0.662-0.975) | 0.025 | 0.752(0.603---0.943) | **0.013** |
| **Blood type** |  |  |  |  |
| O | reference |  |  |  |
| A | 0.883(0.732---1.065) | 0.196 |  |  |
| B | 0.948(0.779---1.153) | 0.6 |  |  |
| AB | 1.028(0.781---1.340) | 0.838 |  |  |
| missing data | 0.883(0.452---1.577) | 0.694 |  |  |
| **CEA** |  |  |  |  |
| normal | reference |  | reference |  |
| high | 5.139(4.333---6.120) | ＜0.001 | 2.948(2.428---3.589) | ＜0.001 |
| missing data | 1.555(0.537---3.575) | 0.35 | 1.835(0.427---8.401) | 0.412 |
| **CA199** |  |  |  |  |
| normal | reference |  | reference |  |
| high | 6.272(5.344---7.364) | ＜0.001 | 3.423(2.845---4.118) | ＜0.001 |
| missing data | 1.056(0.492---1.995) | 0.876 | 3.384(0.623---17.019) | 0.144 |
| **AFP** |  |  |  |  |
| normal | reference |  | reference |  |
| high | 1.166(0.180---4.355) | 0.8411 | 1.035(0.150---4.368) | 0.966 |
| missing data | 0.468(0.229---0.848) | 0.022 | 0.257(0.063---0.866) | 0.043 |
| **Tumor location** |  |  |  |  |
| right colon | reference |  |  |  |
| left colon | 0.911(0.744---1.116) | 0.37 | 0.906(0.717---1.143) | 0.407 |
| rectum | 0.691(0.577---0.829) | ＜0.001 | 0.927(0.747---1.154) | 0.501 |
| **Tumor size (cm)** |  |  |  |  |
| ≤3 | reference |  | reference |  |
| >3 | 2.390(1.967---2.927) | ＜0.001 | 1.555(1.238---1.964) | ＜0.001 |
| missing data | 7.215(3.000---16.339) | ＜0.001 | 2.017(0.703---5.550) | 0.18 |
| **Pathological type** |  |  |  |  |
| adenocarcinoma | reference |  | reference |  |
| others | 0.452(0.337---0.594) | ＜0.001 | 0.347(0.251---0.470) | ＜0.001 |
| **Differentiation** |  |  |  |  |
| G1-G2 | reference |  | reference |  |
| G3-G4 | 0.503(0.386---0.664) | ＜0.001 | 0.777(0.566---1.079) | 0.126 |
| missing data | 1.434(0.997---2.068) | 0.052 | 0.431(0.254---0.725) | ＜0.001 |
| **Invasion depth** |  |  |  |  |
| T1-T2 | reference |  | reference |  |
| T3-T4 | 1.920(1.565---2.373) | ＜0.001 | 1.139(0.906---1.441) | 0.268896 |
| missing data | 13.011(9.909---17.161) | ＜0.001 | 9.157(6.085---13.934) | ＜0.001 |
| **HBsAg** |  |  |  |  |
| - | reference |  | reference |  |
| + | 0.689(0.494---0.939) | 0.023 | 0.682(0.473---0.961) | **0.034** |

^#^ other types: carcinoid, signet ring cell carcinoma , mucinous adenocarcinoma, etc.. P＜0.05 was statistically significant.

**Supplementary table 2. Influence of HBsAg on synchronous liver metastases in early-onset CRC**

| **Parameters** | **Synchronous lung metastases, N (%)** | **No synchronous lung metastases, N (%)** | **Total** | **χ^2^** | **P value** |
| --- | --- | --- | --- | --- | --- |
| **HBsAg** |  |  |  | 2.583 | 0.108 |
| + | 13（10.7%） | 109(89.3%) | 122 |  |  |
| - | 136(16.3%) | 698(83.7%) | 834 |  |  |
| **Total** | 149（15.6%） | 807（84.4%） | 956 |  |  |

P＜0.05 was statistically significant.

**Supplementary table 3. Influence of e-antigen, liver cirrhosis index and virus carrier status on liver metastasis in early-onset CRC with HBsAg(+) group**

| **Group** | **Synchronous liver metastases, N (%)** | **No synchronous liver metastases, N (%)** | **P value** |
| --- | --- | --- | --- |
| **HBeAg** |  |  | 1.000 |
| + | 2(10.0%) | 18(90.0%) |  |
| - | 11(10.8%) | 91(89.2%) |  |
| **APRI** |  |  | 1.000 |
| APRI high level | 2（8.7%） | 21（91.3%） |  |
| APRI low level | 11（11.1%） | 88（88.9%） |  |
| **FIB-4** |  |  | 0.790 |
| FIB-4 high level | 2(7.4%) | 25（92.6%） |  |
| FIB-4 low level | 11（11.6%） | 84（88.41%） |  |
| **Virus carrier status** |  |  | 0.050 |
| HBsAg/HBeAg/HBcAb(+) | 2（10.5%） | 17（89.5%） |  |
| HBsAg/HBeAb/HBcAb(+) | 7(7.8%) | 83(92.2%) |  |
| Unknown | 4(30.8%) | 9(69.2%) |  |

P＜0.05 was statistically significant.

**Supplementary table 4. Influence of HBsAg on synchronous liver metastases in colon cancer**

| **Parameters** | **Synchronous lung metastases, N (%)** | **No synchronous lung metastases, N (%)** | **Total** | **χ^2^** | **P value** |
| --- | --- | --- | --- | --- | --- |
| **HBsAg** |  |  |  | 0.149 | 0.700 |
| + | 32（14.3%） | 191(85.7%) | 223 |  |  |
| - | 410(15.3%) | 2267(84.7%) | 2677 |  |  |
| **Total** | 442（15.2%） | 2458（84.8%） | 2900 |  |  |

P＜0.05 was statistically significant.

**Supplementary table 5. In the colon cancer with HBsAg+ group, the effect of e antigen, liver cirrhosis index and virus carrier status on liver metastasis**

| **Group** | **Synchronous liver metastases, N (%)** | **No synchronous liver metastases, N (%)** | **P value** |
| --- | --- | --- | --- |
| **HBeAg** |  |  | 0.581 |
| + | 5(20.0%) | 20(80.0%) |  |
| - | 27(13.6%) | 171(86.4%) |  |
| **APRI** |  |  | 1.000 |
| APRI high level | 4（13.3%） | 26（86.7%） |  |
| APRI low level | 28（14.5%） | 165（85.5%） |  |
| **FIB-4** |  |  | 0.889 |
| FIB-4 high level | 15(14.7%) | 87（85.3%） |  |
| FIB-4 low level | 17（14.0%） | 104（86.0%） |  |
| **Virus carrier status** |  |  | 0.134 |
| HBsAg/HBeAg/HBcAb(+) | 5（22.7%） | 17（77.3%） |  |
| HBsAg/HBeAb/HBcAb(+) | 22(12.2%) | 159(87.8%) |  |
| Unknown | 5(25.0%) | 15(75.0%) |  |

P＜0.05 was statistically significant.
